# PART is part of Alzheimer disease

**DOI:** 10.1007/s00401-015-1390-7

**Published:** 2015-01-28

**Authors:** Charles Duyckaerts, Heiko Braak, Jean-Pierre Brion, Luc Buée, Kelly Del Tredici, Michel Goedert, Glenda Halliday, Manuela Neumann, Maria Grazia Spillantini, Markus Tolnay, Toshiki Uchihara

**Affiliations:** 1 Laboratoire de Neuropathologie Escourolle, AP-HP, Hôpital de la Salpêtrière, 47 Bd de l’Hôpital, 75651 Paris Cedex 13, France; 2 Alzheimer Prion Team, Inserm U 1127, CNRS, UMR 7225, Sorbonne universités, UPMC, UMR S 1127, ICM, 47 Bd de l’Hôpital, 75651 Paris Cedex 13, France; 3 Clinical Neuroanatomy/Department of Neurology, Center for Biomedical Research, University of Ulm, 89081 Ulm, Germany; 4 Laboratory of Histology, Neuroanatomy, and Neuropathology, ULB Neuroscience Institute (UNI), Université Libre de Bruxelles, 1070 Brussels, Belgium; 5 Inserm-University of Lille, 59045 Lille Cedex, France; 6 MRC Laboratory of Molecular Biology, Francis Crick Avenue, Cambridge, CB2 0QH UK; 7 Neuroscience Research Australia and University of New South Wales, Sydney, 2031 Australia; 8 Department of Neuropathology, University of Tübingen, Calwerstrasse 3, 72072 Tübingen, Germany; 9 German Center for Neurodegenerative Diseases (DZNE), 53175 Bonn, Germany; 10 Department of Clinical Neurosciences, University of Cambridge, The Clifford Allbutt Building, Cambridge, CB2 0AH UK; 11 Department of Neuropathology, Institute of Pathology, University Hospital, 4031 Basel, Switzerland; 12 Laboratory of Structural Neuropathology, Tokyo Metropolitan Institute of Medical Science, Kamikitazawa, Setagaya, Japan

**Keywords:** Alzheimer disease, Amyloid, Aβ, PART hypothesis, Tau, Tauopathy

## Abstract

It has been proposed that tau aggregation confined to entorhinal cortex and hippocampus, with no or only minimal Aβ deposition, should be considered as a ‘primary age-related tauopathy’ (PART) that is not integral to the *continuum* of sporadic Alzheimer disease (AD). Here, we examine the evidence that PART has a pathogenic mechanism and a prognosis which differ from those of AD. We contend that no specific property of the entorhinal–hippocampal tau pathology makes it possible to predict either a limited progression or the development of AD, and that biochemical differences await an evidence base. On the other hand, entorhinal–hippocampal tau pathology is an invariant feature of AD and is always associated with its development. Rather than creating a separate disease entity, we recommend the continued use of an analytical approach based on NFT stages and Aβ phases with no inference about hypothetical disease processes.

## Introduction

Tau aggregation in nerve cell bodies (neurofibrillary tangles, NFTs) and neurites (neuropil threads and the *coronae* of neuritic plaques) constitutes the tau pathology of Alzheimer disease (AD). Aggregated Aβ accumulates extracellularly as diffuse or focal deposits. The severity of AD-related tau and Aβ pathologies is routinely evaluated by their distribution patterns in the brain in addition to the density of neuritic plaques (ABC score [17, 22]). Tau pathology, in the context of AD, follows a hierarchical distribution, occurring in ordered sets of regions: It can be found in the entorhinal cortex alone, in the entorhinal cortex and the hippocampus, or in the entorhinal cortex, the hippocampus, and the neocortex [1]. Aβ deposits can be found in the neocortex alone, in the neocortex and the hippocampus, or, in addition, in the basal ganglia, the mesencephalon, and the cerebellum [26]. The number of areas that are involved in tau and Aβ pathology increases continually in a definite sequence. This observation indicates that the accumulation, be it of tau or of Aβ, is not or only minimally cleared. It also implies a progression independent of lesion density. This is the basis of the neurofibrillary tangle (NFT) stages (assessing tau pathology) [1, 2] and of the Aβ phases (assessing Aβ deposits) [26].

Crary et al. [8]. suggest creating a separate neurodegenerative disease called PART (‘primary age-associated tauopathy’) that describes cases with tau pathology in the entorhinal cortex and hippocampus (ECH tau pathology) either without Aβ deposits (tau+/Aβ-) or with minimal Aβ deposits. The number of Aβ deposits that is compatible with PART and the techniques used to detect them remain to be determined. It is proposed that such topographically limited tau pathology is ‘age-related’ but unrelated to AD. These cases are characterized by a low NFT stage (I–III or IV) with no or little Aβ deposition (Aβ phase 0 to 2), or tau/Aβ I/0, tau/Aβ II/0, etc., with the first score here signifying the NFT stage, the second the Aβ phase, and tau/Aβ as the abbreviation for tau pathology/Aβ deposits.

## PART and ECH tau pathology

There is consensus regarding the high prevalence of tau pathology in the ECH without amyloid deposition (tau/Aβ I/0, II/0, III/0, and, rarely, IV/0). Two main hypotheses currently exist for integrating such cases nosologically: One favors unity, i.e., a *continuum* from tau+/Aβ− to tau+/Aβ+; the PART concept posits a duality of processes, AD vs ‘aging’, with age-associated tauopathy (tau+/Aβ-) defining PART and considered to be a process different from AD-associated tau+/Aβ+.

According to the PART hypothesis, tau+/Aβ- cases have a condition that differs from AD and would not have reached NFT stages higher than IV and Aβ phases greater than 2, even if they had lived longer. According to the same hypothesis, tau pathology is associated with aging, whereas Aβ deposition is linked to AD with the inference that very old people should be spared by the latter and affected by the former. If tau pathology is indeed constant in centenarians, so too is Aβ deposition [10]––an observation that contradicts the supposed distinction between aging and AD pathology.

In view of the new possibilities for imaging tau pathology and Aβ deposits, it is probable that advances in neuroimaging will enable us to answer, in the future, the question of the unity or duality of these pathologies. Very elderly people will have to be included in such studies to cope with the long duration, and possibly uneven progression, of tau and Aβ pathologies. A distinction will have to be made between the increase in the local density of the lesions with age and the increase in the number of affected regions. The prognostic value of quantified ECH tau pathology across the aging spectrum will be a major issue. According to the PART hypothesis, tau pathology in the ECH, when it is not accompanied by Aβ deposition, will not progress (or is less likely to progress). Alternatively, if the *continuum* hypothesis is correct, the risk of developing AD will be significantly increased in cases with ECH tau pathology, and ECH tau pathology will always precede full-blown AD within a time period that remains to be determined. From a mechanistic point of view, the PART and tau *continuum* hypotheses suggest fundamental differences. If ECH tau pathology, which, according to the PART hypothesis, is not integral to AD, is in fact part of the early stages of AD, then the amyloid cascade hypothesis will have to be amended.

The *continuum* hypothesis does not exclude the possibility that a specific tauopathy affects the hippocampus with particular severity and causes marked neuronal loss at a stage where the number of involved regions is limited (local severity with limited extension). Such cases should be detected in the future by comparing the local severity of the lesions in the hippocampus with the NFT stage––this comparison is not performed in the current PART criteria. Such cases, which at first approximation may be rare, are confused with early AD cases using the proposed PART criteria: Indeed, most of the tau+/Aβ- cases appear to be a stage of AD rather than a specific form of disease, according to the *continuum* hypothesis that is developed here.

## PART and SNAP

The attempt to isolate PART, with its combination of changes (tau+/Aβ−) differing from the combinations tau−/Aβ+ and tau+/Aβ+ thought to characterize AD, is reminiscent of the recent classification, based on biomarker profiles, of cases with normal Aβ biomarker values but with “brain injury biomarkers” or “neuronal injury biomarkers” (including elevated CSF tau and phospho-tau), a combination that does not fit the anticipated progression, i.e., an abnormal level of CSF Aβ (stage 1), associated with brain injury biomarkers (stage 2), and, finally, with subtle cognitive impairment (stage 3) [21, 25]. These cases have been assigned to the “suspected non-Alzheimer pathophysiology” (SNAP) group. One is inclined to equate SNAP (brain injury+/Aβ- biomarkers) and PART (tau+ and Aβ− pathologies at post-mortem)––as recently discussed [19]. It is beyond the scope of this paper to address the question (the interested reader is referred to [19]), but the reasons leading to the isolation of SNAP and PART are strikingly similar. We will confine our discussion to the sequence of tau and Aβ pathologies in AD and to the relevance of isolating PART as a specific entity. Some of the conclusions drawn here regarding PART, however, could apply in the future to SNAP: the sequence of biomarker changes proposed by Jack in SNAP, for instance, is also relevant to PART [19].

## Possible benefits of the PART hypothesis

Several reasons have been proposed for isolating PART from the *continuum* of AD. The first rests upon practical advantages. PART can facilitate communication among pathologists, clinicians, and researchers; it can separate pathologic classification from clinical diagnosis; and it can also help neuropathologists to avoid using the term dementia. These reasons are not compelling. Crary et al. [8] believe that PART is sufficient to span the tau/Aβ indices from I/0 to IV/0, but the term has the obvious disadvantage of a loss of information: The stages and phases reflect greater diversity than can be subsumed under the PART umbrella. On the other hand, adding the PART label to the tau/Aβ indices I or II/0, 1 or 2, as recommended by Crary et al., does not add any information because PART is used synonymously with the tau/Aβ indices. The term PART simply implies that the neuropathologist takes an option about the putative prognosis and pathogenesis of these indices. The current practice of NFT stages and Aβ phases already separates pathology from clinical diagnosis and prevents the neuropathologist from using the clinical term of dementia in his/her diagnoses: NFT stages and Aβ phases are, in fact, staging procedures and not diagnostic criteria.

Additional reasons that pertain to the question of the existence of a specific pathology different from that of AD have been put forward [8]:

1. The average age at death is generally higher for PART patients than for those with AD.
2. There is no association of PART with the APOEε4 allele. An association with the tau H1 haplotype has been found in ‘tangle-predominant’ forms of AD, which are now subsumed under the PART umbrella.
3. The more severe PART pathology is associated with a higher age at death and lower scores on cognitive tests.

These points will be discussed later. We contend first that there is no way, neuropathologically, genetically, or clinically, to differentiate PART from early AD.

## No characteristics of the tau pathology permit the independent diagnosis of PART from preclinical/early AD

**Fig. 1:**
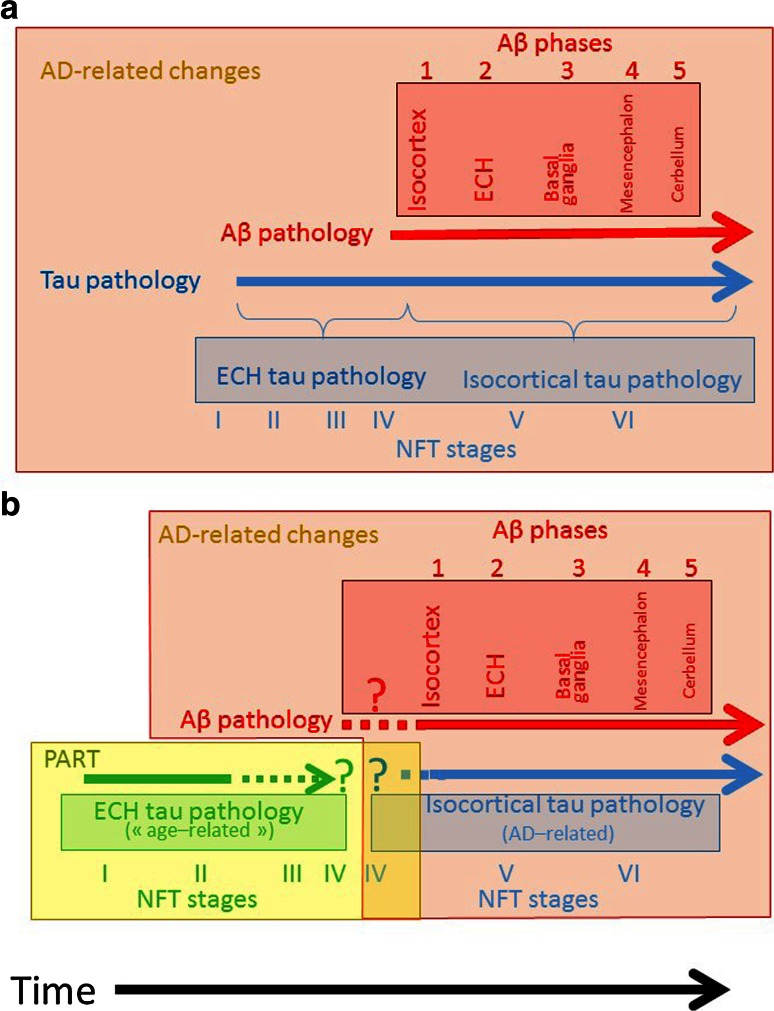
Relationship between the development of tau pathology and Aβ deposition. **a** Tau pathology in the entorhinal cortex and hippocampus (ECH) belongs to the AD continuum. It is complemented over time by Aβ deposition that occurs in an ordered manner (no attempt has been made to represent the duration of each stage or phase; only the sequence is important here). **b** *Panel* b illustrates the PART hypothesis and the issues it raises. Tau pathology in the ECH differs from tau pathology in AD, with three major problems indicated by the dotted lines. *1* Can Aβ deposition precede tau pathology? *2* Can tau pathology related to PART occur with Aβ deposition phases 1–2? *3* How and where does PART end? Panel a describes our position

The PART hypothesis raises questions that are currently impossible to resolve (Fig. 1). In the *continuum* hypothesis, tau pathology begins in the ECH (NFT stages I to III or IV) in the absence of Aβ deposits (Aβ phase 0); tau pathology is later found in the isocortex (NFT stages V and VI) together with Aβ deposits (Aβ phase 1 or higher). Tau deposition in the ECH is necessary but not sufficient for the development of AD.

The intrinsic properties of tau aggregation are identical in the early and late NFT stages. According to the PART hypothesis, ‘age-related’ medial temporal lobe NFTs – occurring in the absence of Aβ deposition––differ from AD-related NFTs––associated with Aβ deposition. The attribution of different diagnoses to the same inclusions (ECH tau pathology) based upon extrinsic characteristics (presence or absence of AD-associated Aβ deposits) could well be less effective than regrouping under the same term similar inclusions that have different extrinsic characteristics, as has recently been done for “tauopathies,” “synucleinopathies,” or “fronto-temporal lobar degeneration-TDP-type.” Moreover, the extrinsic characteristics that permit the diagnosis of PART raise questions that two hypothetical examples will help to illustrate.

### AD at onset cannot be distinguished from PART with a low number of Aβ deposits

In the first example, AD develops in a patient who was not previously affected by PART. Will the Aβ deposits first be found in isolation (NFT stage 0, Aβ phases 1 or higher)? In our experience, isolated Aβ deposition (without any tau pathology) is exceptional in large cohorts in which tau aggregation and Aβ deposition have been systematically studied [3], whereas tau deposition in ECH is the most common pathology with increasing age. We are surprised by the high prevalence of ‘low,’ ‘moderate,’ or even ‘high’ plaque scores at NFT stage 0 in Crary et al. [8]. As a rule, some tau pathology is found in the ECH when Aβ is found in the isocortex. How will that situation (NFT stages I, II, III/Aβ phases 1 or higher), occurring at the onset of AD, be distinguished from PART? Or does AD (in contrast to PART) induce a tauopathy that involves at once the isocortex and the ECH without progression––with the unexpected consequence that low NFT stages should always be considered to be age-related?

### PART with AD cannot be distinguished from AD

In the second example, a patient develops PART and, later, AD. An “age-related” tauopathy is initially found in the ECH (NFT stages I, II, or III). At the onset of AD, Aβ deposits appear in the isocortex (Aβ phase 1). Does the “age-related” tauopathy that was initially present in ECH still qualify as PART as soon as Aβ deposition occurs, or does it become AD-related since it loses its status of a “pure tauopathy,” which defines PART? The logic would be to consider, despite the term “pure,” that PART remains PART even when AD starts to develop. Should it be considered then that ECH tau pathology is mixed (AD- and “age-related”), but that their components cannot be separated because there is, at least currently, no way to distinguish between AD and age-related tau aggregation? Should it be considered, on the contrary, that tau pathology, inasmuch as it involves the ECH, always belongs to PART with the unexpected consequence of excluding from AD pathology the lesions in the hippocampus that continue to worsen during the course of AD? As seen from the examples above, neither the topography nor the intrinsic properties of the tauopathy enable one to separate “age-related” and AD-related processes.

## In the elderly, Aβ deposition is linearly proportional to NFT stages, with no inflection caused by PART

**Fig. 2:**
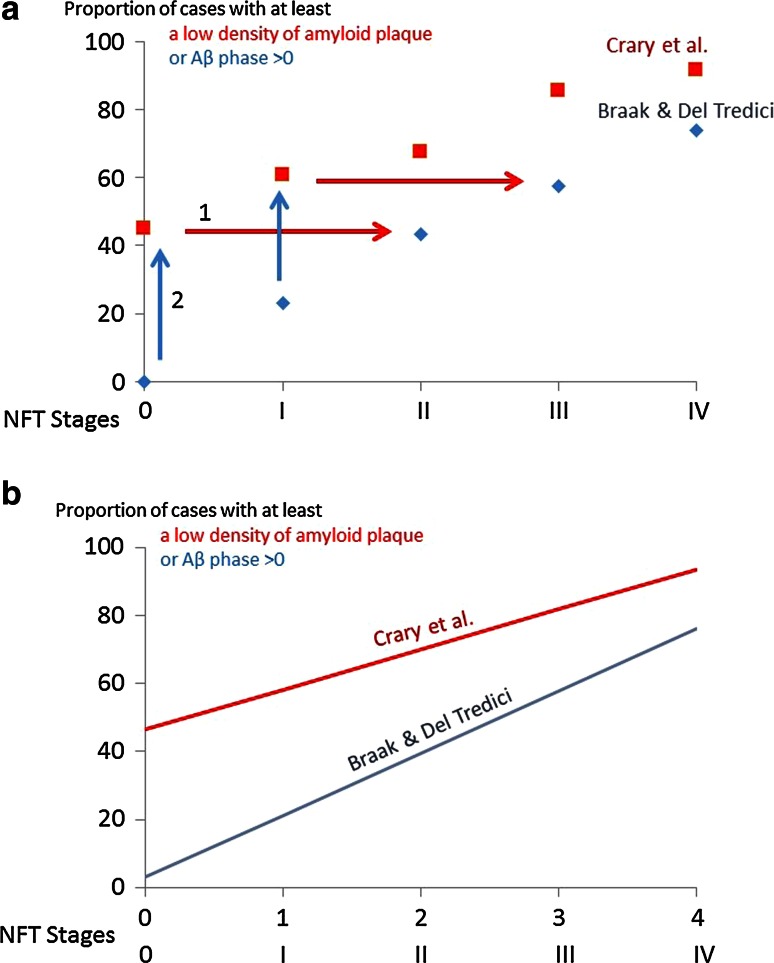
Linear relationship between the NFT stage and the proportion of cases with Aβ pathology. **a** Raw data from Crary et al. [8] (*red squares*) and Braak and Del Tredici [5] (*blue diamonds*). The *red squares* indicate the percentage of cases with ‘low,’ ‘moderate,’ or ‘high’ amyloid plaque score. The *blue diamonds* indicate the percentage of cases with Aβ phases 1, 2, 3, 4 or 5. *Red squares* and *blue diamonds* thus refer to the cases with Aβ deposition, whatever its severity. The *blue arrows* indicate the translation along the *Y* axis that should be applied to Braak and Del Tredici data [5] so that they coincide with the Crary et al. data [8]. The *red arrows* indicate, alternatively, the translation that should be applied along the *X* axis so that the Crary et al. data [8] coincide with Braak and Del Tredici data [5] (see text for explanation). **b** Regression line between the NFT stages and the proportion of cases with Aβ pathology. To compute the correlation coefficient, the slope and the intercept of the regression lines, the NFT stages (I to IV) had to be translated into numerical value (1–4). Equation of the *red line* (data from Crary et al. [8]): Proportion of cases (in percentage) with low, moderate or high plaque score = 11.78 × NFT stage + 46.5. The correlation coefficient *r* = 0.99 indicates that the NFT value suffices to predict with a high degree of accuracy the proportion of cases with Aβ pathology. Equation of the *blue line* (data from Braak and Del Tredici) [5]: Proportion of cases (in percentage) with Aβ phase higher than 0 = 18.21 × NFT stage + 3.11. The correlation coefficient *r* = 0.995 indicates that the NFT stages almost fully predicts the proportion of cases with Aβ pathology

Once cases harboring genes that enhance early Aβ deposition are excluded from the population, the number of cases with Aβ accumulation and signs of ‘neurodegeneration’ is linearly proportional to age in vivo [20]. The difficulties in separating ‘age-related’ from AD-related processes are illustrated by Table 1 from Crary et al. [8] in which cases with Aβ deposition in the cortex (phase 1) or in the hippocampus (phase 2)––i.e., cases with tau/Aβ I/1 or III/2—are classified as Pure Age Related Tauopathy (PART), a contradiction in terms. According to the PART hypothesis, the presence of Aβ deposits speaks for the presence of an AD-related process rather than a pure tauopathy. Biochemical analysis of the isocortex has revealed, however, the presence of Aβ aggregates that were not detected by immunohistochemistry at low NFT stages [9]. A significant proportion of PART cases in Table 1 of Crary et al. [8] exhibits a low-plaque score considered compatible with possible PART––up to 35 % of the cases in NFT stage II. But is it justified to separate the ‘low’ plaque scores as compatible with possible PART from the ‘moderate’ and ‘high’ scores that would be related to AD? The pooled prevalence of cases with ‘low,’ ‘moderate,’ and ‘numerous’ amyloid plaque scores in Table 1 of Crary et al. [8], with no distinction of possible PART cases from the whole population, appears to be strongly linked with the NFT stage. The correlation between the NFT stage and the proportion of cases with at least a low amyloid plaque score in Crary et al. [8] (*r* = 0.989) or Aβ deposition at least at phase 1 in Braak and Del Tredici [4] (*r* = 0.995) is close to 1––the high value of r indicating that it is possible to predict with a high degree of precision the proportion of cases with Aβ pathology only from the value of the NFT stage (Fig. 2). The probability of having the cases with AD and the cases with PART aligned if two independent processes were to be involved is extremely low. If there were to be two pathogenic processes, the slopes and intercepts should have been different at low (supposedly falling under the diagnosis of PART) and high (supposedly falling under the diagnosis of AD) NFT stages.

Correlation is not causality: It may be taken as evidence of Aβ pathology causing or promoting tau pathology, of tau pathology causing or promoting the development of Aβ pathology, or of a third (currently unknown) variable responsible for the synchronous development of the two pathologies. However, the comparison of the two curves linking the proportion of cases with Aβ pathology and NFT stage shows a remarkable difference between the data of Crary et al. [8]. and of Braak and Del Tredici [5]: In the first dataset, a high proportion of NFT 0 cases have Aβ plaques, whereas in the second dataset there is no case with Aβ that has no tau pathology (Fig. 2a). Moreover, the slopes are, although not identical, very similar (Fig. 2b). The difference in datasets may, in our view, be given two opposing interpretations: Either Crary et al. do not detect NFT stages I and II and the curve representing their data has to be translated along the X-axis (red arrows in Fig. 2a), or Braak and Del Tredici do not detect Aβ deposits with as high a sensitivity as Crary et al. and the curve representing their dataset has to be translated along the *Y* axis (blue arrows in Fig. 2a). We favor the first explanation: The ‘amyloid plaque score’ was obtained with different methods in the cohort of Crary et al. [8]. Using thin sections, while thick sections with larger areas were examined using a sensitive silver method (Campbell–Switzer) in the cohort of Braak and Del Tredici [5]. On the other hand, the tissue sample should include the entorhinal cortex, i.e., must involve the most anterior portion of the hippocampus, to detect NFT stages I and II accurately [1]—a sample not commonly used in routine practice––and, finally, AT8 immunohistochemistry (one of the most sensitive techniques for detecting tau pathology used by Braak and Del Tredici [5]) is not mandatory in the NIA-AA criteria [17, 22]. It should be added that the debate could be resolved by an exchange of slides from early stages and phases. The question of the precedence of tau or of Aβ pathology could have important implications for the identification of therapeutic targets.

**Fig. 3:**
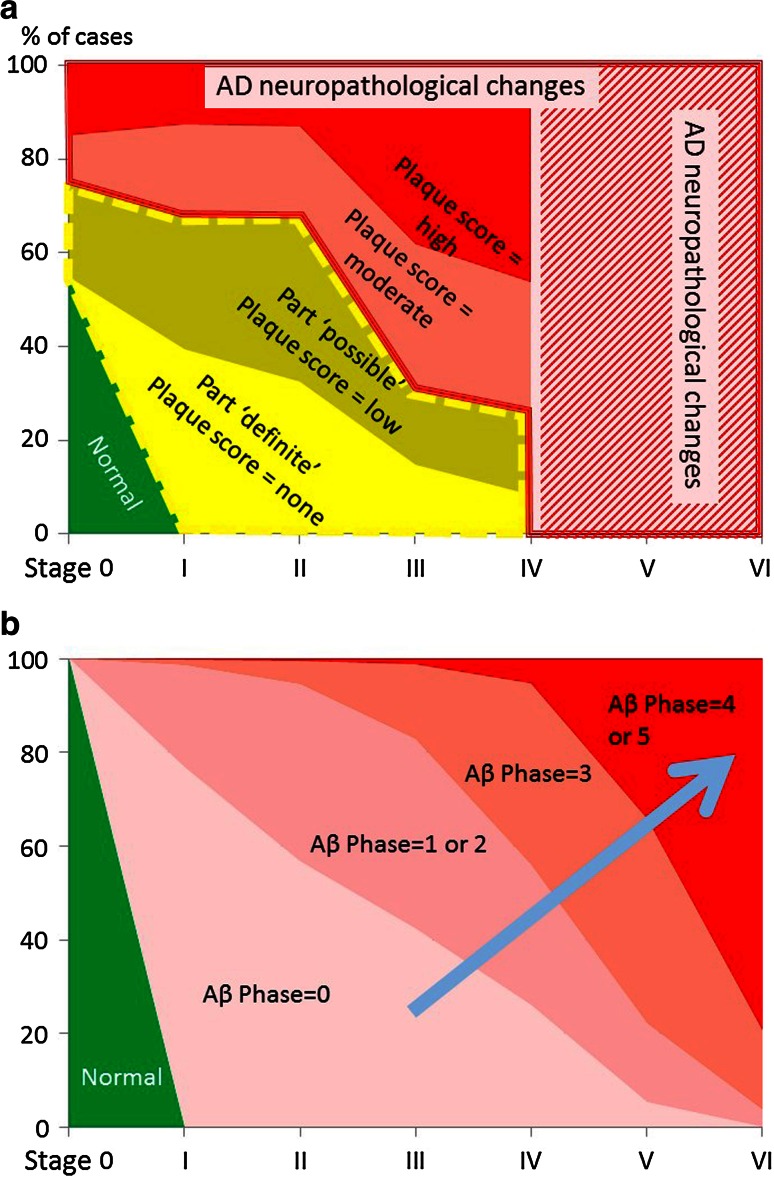
Relationship between the NFT stage and the proportion of the population with an ‘amyloid plaque score’ of ‘none’ (PART definite), ‘low’ (PART possible), and ‘high’ (‘AD neuropathologic changes’). Comparisons with NFT stages and Aβ phases. Area chart. The percentage of the population (*y* value) corresponding to the NFT stage (*X* axis) for each category (color and label shown on each area) is proportional to the length of its projection on the *Y* axis. **a** The values shown here have been calculated from Table 1 of Crary et al. [8]. Percentage of normal cases (*green*) = NFT stage 0, amyloid plaque score = none. The PART area is surrounded by a *dotted yellow line*. Percentage of cases with PART ‘definite’ (*yellow*) = NFT stage > 0, amyloid plaque score = none. Percentage of cases with PART ‘possible ‘(*dark yellow*) = NFT stage > 0; amyloid plaque score = low. AD neuropathological changes [22] are indicated in *shades of red*, with a *red border*. The *red* and *white cross-hatching* corresponds to an area extrapolated from Table 1 of Crary et al. [8]: The diagnosis is necessarily AD neuropathological changes because NFT stages are >4 with a ‘moderate’ or ‘high’ plaque score. The *blue arrow* indicates the progression of Alzheimer disease neuropathological changes as we understand them in the context of the PART hypothesis: PART cases do not commonly progress to Alzheimer disease. Alzheimer disease begins with a low plaque count (or low Aβ phase) in the absence of tau pathology in the ECH. Please note that the frequency of cases with NFT stage 0 and ‘amyloid plaques’ (up to ‘numerous’ plaque score) is low in our experience (see text and [3]). It should also be emphasized that the dividing line between PART ‘definite’ and ‘possible’, and between PART ‘possible’ and AD-related changes is in Table 1 of Crary et al. [8] relies on an “amyloid plaque score” that differs from the “neuritic plaque score” recommended in the NIA–AA criteria [22] and from the Aβ phases recommended in the PART diagnostic criteria proposed in Table 2 of Crary et al. [8]. The difference between the density of neuritic plaques and of all types of Aβ deposits may be considerable. **b** The values shown here have been calculated from the cohort presented in Braak and Del Tredici [4]. Normal (*green*) = NFT stage 0, plaque score = none. The proportion of cases in the observed combination of NFT stages and Aβ phases are indicated in *red* *shading*. The *blue arrow* indicates the progression of the changes in the context of the *continuum* hypothesis defended here

In the final analysis, the relationship between the proportion of cases with Aβ pathology and the NFT stage here is linear. No inflection in the curve suggests the occurrence of different pathogenic processes, such as PART, that would change the slope of the curve [8]. The percentage of ‘pure’ PART cases thus decreases linearly with the NFT stage, a proportionality that argues in favor of a *continuum* between tau+/Aβ− cases and tau+/Aβ+ cases. The most succinct explanation for the distribution of tau and Aβ pathologies in AD is a progression that begins with tau pathology in the ECH, followed by Aβ deposition in the isocortex, and ends with the gradual propagation of tau pathology to the isocortex. The time course of this progression is slow and cannot be predicted at present. The PART hypothesis artificially divides the progression (see Fig. 3).

## No clinical or genetic characteristics permit the differentiation of PART from preclinical/early AD

Crary et al. [8] suggest that patients with PART have special genetic characteristics. We think that the dataset presented by Crary et al. [8] are not convincing in that respect and can be interpreted within the framework of a *continuum* from tau/Aβ I/0 to tau/Aβ VI/5. Since the number of cases per diagnostic category in Table 1 of Crary et al. [8] is small, conclusions should be drawn with caution. With this caveat in mind, the statement that the age at death for patients with PART is higher than for those with AD is not supported by Table 1 of Crary et al. [8]. Comparisons were made there between NFT stage 0 and the other stages (the mean age of death did indeed increase with stage), but comparisons were not made within the columns for each stage between the cases with and without Aβ deposits. The differences were small, and usually––but not always––indicated a younger age of the patients with Aβ deposits, but this could have been driven by the ApoE ε4 allele.

Crary et al. [8] claim that “there is an absence of an association between PART and the strongest risk factor for AD, the APOE ε4 allele,” but the data supplied in their Table 1 do not support this statement. Instead, they indicate that the ApoE ε4 allele is more frequent in PART ‘definite’ cases (amyloid plaque density = none) at NFT stages I, III, and IV than in normal cases at NFT stage 0. Although not statistically significant, these results indicate that even definite PART cases do not show any tendency towards a lower ApoE ε4 frequency. In line with this, the frequency of the APOE ε4 allele had previously been reported to be higher in NFT stage I cases than in stage 0 cases in a cohort of patients in which the comparison reached significance, suggesting an influence of ApoE ε4 even on the development of early tau pathology [14]. Cases with PART ‘possible’ at NFT stages IV have, again, a higher ApoE ε4 allele frequency than cases with low AD-related changes (i.e., low amyloid plaque density and NFT stage 0), and the value reaches significance if we understand the legend accompanying Table 1 of Crary et al. that states that all comparisons were made with “Braak NFT stage 0 cases” (although one value labeled as significant is in the Braak NFT stage 0 column) [8].

The data in the literature concerning the H1 haplotype of *MAPT,* the tau gene, indicate multiple effects. It is well established that H1 is associated with progressive supranuclear palsy (PSP) and corticobasal degeneration (CBD), two diseases with tau inclusions, as well as with Parkinson disease [13]. It has also been reported that the H1c haplotype increases the risk of AD [24]. Tau pathology and the density of Aβ plaques are less severe with the H1 tau haplotype, both in early- and in late-stage cases of AD [23, 27]. Current data thus indicate that the H1 haplotype modulates the risk of AD as well as the severity of the pathology, but there is no evidence that it can be taken as a specific marker of PART. Of course, larger datasets and agreement on comparator groups are needed to ascertain any differences in genotype between different types of cohorts, whether those considered to be progressing along a *continuum*, or those considered as having dichotomous disease processes.

## PART and experimental seeding/spreading of tau pathology

Arguments for and against each hypothesis will probably remain unsatisfactory in the absence of a pathophysiological mechanism. Why are Aβ and tau pathologies intermingled and what is their causal relationship? Why do they progress? It should be emphasized that, experimentally, the progression of tau pathology in a prion-like manner is not confined to the ECH [6, 7, 12, 15, 18], nor does the spreading of aggregated tau require the presence of Aβ deposits. The involvement of the brainstem and the spinal cord has been observed at NFT stage I [11, 16]. It has to be determined which differences, molecular or otherwise, could explain why the tangles of PART should remain confined to the ECH, whereas the tangles of AD would be capable of inducing the spread of tau pathology in the brainstem, spinal cord, and neocortex.

In conclusion, both Aβ and tau pathologies are necessary for the diagnosis of AD according to current criteria [17, 22]. However, neuropathological data, presented in this paper, support the view that at the early phase only the tau component may be apparent. Tau deposition is a necessary but not sufficient pathology for the development of AD. We support the continued use of an assessment of NFT stages and Aβ phases (tau/Aβ), to which the CERAD score may be added [17, 22], without inference about the disease the patient would have developed if s/he had lived longer. This will facilitate correlations between clinical data, including biomarkers and neuroimaging, and pathology. We believe that the PART hypothesis does not add to the pathological description and may be confusing. It would be an improvement if it were to have a prognostic value and if it could be demonstrated that PART arises through a pathogenic mechanism distinct from that of AD. At present, there is no evidence that PART and AD are the result of two entirely different processes.
